# High prevalence of metal detector activation and its psychological impact in patients with total knee arthroplasty: A case‐control study

**DOI:** 10.1002/jeo2.70572

**Published:** 2025-11-28

**Authors:** Jérôme Murgier, Etienne Cavaignac, Régis Pailhé

**Affiliations:** ^1^ Clinique Aguiléra – Ramsay Santé Biarritz France; ^2^ Clinique Universitaire du Sport CHU Toulouse Toulouse France

**Keywords:** airport screening, airport security, cobalt‐chromium, implant safety, metal detection, patient experience, security protocols, titanium, TKA implants, total knee arthroplasty

## Abstract

**Purpose:**

Metal detectors in airports are designed to identify metallic objects on passengers, which can inadvertently trigger alarms for patients with total knee arthroplasty (TKA) implants. The main goal of this study was to compare the prevalence of metal detection at security airport between a cohort of patient who sustained a TKA and another group free of any metallic implants. The hypothesis was that patients with TKA would trigger metal detectors more frequently than those without TKA and this would have a detrimental impact on their whole postsurgical experience.

**Methods:**

A continuous, nonrandomised, case‐control, cross‐sectional study was carried out. Patients who underwent a TKA between 2020 and 2021 at the same hospital were included in this study. A control group was included to compare the results and improve the level of evidence. This cohort was recruited via patient fellows who accompanied them in clinics.

**Results:**

A total of 101 patients were included, 51 patients in the TKA group (over 64 patients) set off the metal detector (79.7%, 95% confidence interval [CI]: 68.3–87.7) versus 5 in the control group (over 37 patients), (13.5%, 95% CI: 5.9–28.0). This difference was statistically significant (*p* < 0.0001). For those who triggered the alarm (51 patients in the TKA group), this event occurred at all security checkpoints they went through for 42 (82.4%) of them whereas in the control group it occurred 20% of the time. This difference was also statistically significant (*p* < 0.05). Additionally, 28 out of the 51 TKA patients who triggered alarms (54.9%) reported that the experience was bothersome and had a negative impact on their mood.

**Conclusion:**

TKA patients more frequently trigger metal detector alarms at airport security checkpoints than patients free of any prosthesis, which cause unnecessary stress and discomfort. As the global number of TKA procedures continues to rise, it is crucial to implement strategies that acknowledge the unique needs of patients with TKA, ensuring both security and comfort during air travel.

**Level of Evidence:**

Level III, retrospective comparative study (cross sectional).

**Abbreviations**
THA: total hip arthroplasty
TKA: total knee arthroplasty

## INTRODUCTION

The prevalence of total knee arthroplasty (TKA) has steadily increased over the past several decades, driven by the ageing population and advancements in surgical techniques [10]. TKA is a highly successful procedure aimed at improving mobility and reducing pain in patients with advanced knee osteoarthritis or other joint degenerative diseases [12]. However, as TKA involves the implantation of metallic components, patients frequently encounter challenges during security screenings at airports and other high‐security areas. Metal detectors, designed to identify concealed metallic objects, can inadvertently detect orthopaedic implants, leading to potential delays, discomfort, and inconvenience for patients [7, 15].

Metal detection systems used in airport security are designed to detect any metallic object that could pose a security risk, such as firearms or explosives. These systems work on the principle of electromagnetic fields, detecting disruptions caused by metallic objects [11]. While most modern metal detectors are calibrated to distinguish between dangerous objects and harmless metal implants [14], individuals with TKA implants often face issues with false alarms, leading to delayed screening, patient disconcertment, and in some cases, unnecessary further security measures [16]. Moreover, advances in airport security systems, such as millimetre‐wave scanners and advanced imaging technologies, have altered how these implants are detected and assessed. Misunderstandings or delays at security checkpoints may contribute to anxiety and stigma associated with medical implants. Despite the importance of this topic, there is a paucity of systematic research exploring the interaction between orthopaedic implants and metal detection systems, leaving clinicians with limited guidance to prepare patients for these situations.

While TKA implants are harmless in terms of flight safety, their interaction with metal detectors has sparked questions regarding patient postsurgical experience and security protocols [8]. It appears feasible that this mounting issue will continue to rise given the increase of annual TKAs globally [17].

The main goal of this study was to compare the prevalence of metal detection at airport security checkpoints between a cohort of patient who sustained a TKA and another group free of any metallic implants.

The hypothesis was that patients with TKA would trigger metal detectors more frequently than those without TKA and this would have a detrimental impact on their whole post‐surgical experience.

## MATERIALS AND METHODS

A continuous, nonrandomised, case‐control study was carried out. Patients who underwent a TKA between 2020 to 2021 at the same hospital were included in this study. They all underwent the same procedure, using the same technique to implant the Stryker triathlon° prosthesis. This implant is primarily composed of titanium (tibial baseplate) and Cobalt‐Chrome (femoral component). These metals are often detected by airport security alarms.

Excluded patients were those who had other metallic implants in their body, whether it was another prosthesis (hip arthroplasty or knee arthroplasty) or an osteosynthesis (nail, plate).

A control group was included to compare the results and improve the level of evidence. This cohort was recruited via patient fellows who accompanied them in clinics. They were asked to confirm that they didn't have any metallic implant in their body.

The minimum follow—up was 2 years and patients were contacted online via the software (‐Orthense°—Digikare and replied to a custom‐made questionnaire. Both groups filled the same questionnaire.

They had to reply to the following questions:

‐ Have you flown since the operation?
‐ If yes, how many times?
‐ Did you trigger the alarm at the airport security checkpoint?
‐ If yes, how many times?
‐ Was it bothersome?

### Statistics

Categorical variables were described as counts and percentage and compared using chi‐square test or exact Fisher test as appropriate. If relevant, two‐sided 95% confidence interval of percentage was computed (Wilson method). Continuous variables were described as median (interquartile range 25%–75%) and compared using Mann–Whitney–Wilcoxon test.

All tests were two‐sided with an alpha risk of 5%.

Statistical analyses were performed using SAS statistical software package, release 9.4 (SAS institute Inc.).

## RESULTS

**Figure 1:**
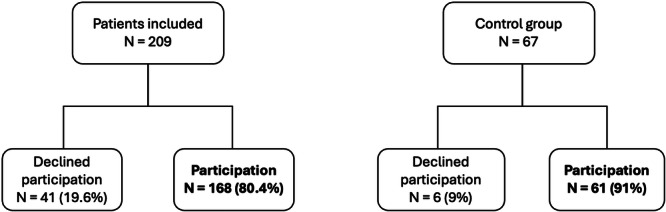
Flow chart.

A total of 209 TKA patients were included, as well as 67 patients in the control group (Figure 1). Some of them (41) declined to participate in this study which resulted in a total of 168 TKA patients and 61 in the control group. Of the 168 TKA patients 64 had flown the last year versus 37 in the control group. Both groups were comparable in terms of demographics (age, sex ratio, body mass index [BMI]); (Table 1) and the mean follow‐up was 18 months (12–26). In the TKA group the average number of flights per patient amounted to 3 (2–5.5) versus 4 (2–6).

**Table 1:** Demographics in the two groups.

	TKA group	Control group	*p* value
Age	65.4 (82–48)	64.7 (77–42)	NS
Sex ratio (M/F)	1.46	1.16	NS
BMI	27.3 (19–38.7)	25.3 (18.6–35)	NS

Abbreviations: BMI, body mass index; TKA, total knee arthoplasty.

Among the 64 TKA patients who had flown, 51 triggered the metal detector (79.7%; 95% CI: 68.3%–87.7%), compared to 5 out of 37 (13.5%; 95% CI: 5.9%–28.0%) in the control group. This difference was statistically significant (*p* < 0.0001, chi‐square test).

Among TKA patients who triggered alarms, 42 (82.4%) reported it occurred at *every* airport screening, whereas this occurred only 20% of the time in controls. This difference was also statistically significant (*p* = 0.0084, Mann–Whitney–Wilcoxon test).

Additionally, 28 out of the 51 TKA patients who triggered alarms (54.9%) reported that the experience was bothersome and had a negative impact on their mood.

## DISCUSSION

The findings of this study clearly indicate that patients who have undergone TKA are significantly more likely to trigger metal detectors during airport security screenings than individuals without metallic implants. Among the TKA cohort, nearly 80% of patients who had flown postoperatively reported triggering alarms, with over 80% of these individuals stating that the event occurred consistently at every checkpoint they encountered. In stark contrast, only 13.5% of individuals in the control group experienced similar events. These results strongly support the initial hypothesis and draw attention to a relatively under‐recognised consequence of joint arthroplasty surgery: the potential for repeated, inconvenient, and sometimes distressing encounters at airport security checkpoints.

Orthopaedic implants such as TKA prostheses are typically composed of dense metallic materials like titanium and cobalt‐chromium alloys, which are known to be easily detectable by conventional metal detection systems. While these implants are entirely benign in terms of security threats, their electromagnetic properties are sufficient to activate standard walk‐through metal detectors. The Stryker® Triathlon prosthesis used in this study includes a cobalt‐chrome femoral component and a titanium tibial baseplate, both of which are highly conductive and thus prone to being detected by electromagnetic field‐based scanning systems [4]. Even with advanced systems, travellers may still undergo secondary screening if a metallic object is detected. Our study suggests that security personnel often do not differentiate effectively between orthopaedic implants and potentially threatening objects, which results in additional screening procedures such as handheld wand scans or pat‐downs. This can cause embarrassment, inconvenience, and anxiety, especially for older patients or those with reduced mobility [6].

Naziri et al. [13] published that most patients with a TKA were setting off the alarm system (83%) whereas Issa et al. [7] found out that this number amounted to 38%. Abbassian et al. [1] reported 71% of their TKA patients triggering the alarm system. This number difference can be related to the relatively small number of patients in Issa's study. Approximately 55% of TKA patients in this study set off the alarms which is relatively comparable to previous studies published on the topic. Interestingly, this number hasn't decreased over time since these three previous studies were published in the 2010's. We could have expected that the new generation of metal detectors would have progressed in selectively detecting harmful objects. When compared to total hip arthroplasties (THA), the results seem similar as Johnson et al. [8] published that 85% of their patients with a THA set off metal detectors at the airport while kimura et al. [9] made the distinction between local airports and international ones. The alarm system was triggered more frequently in international airports (56%) than in local ones (23%). This difference was not obvious in our study.

The second point of this study was to assess the psychological impact of this experience of triggering the alarm and having to go through a second security check most of the time. Most patients in different studies describe the experience as bothersome, citing negative emotional or psychological impacts. Naziri et al. [13] described that 63% of TKA patients reported that the prosthetic joint caused an inconvenience while travelling because of metal detection. This number was smaller in Issa et al. [7] study as it reached 34%. In our study, it amounted to 54.9%, which indicates that more than half are dissatisfied. This finding is particularly important, as it underscores that the implications of orthopaedic implants extend beyond physical recovery and function. Patients may face repeated, stressful interactions while travelling, potentially affecting their willingness to fly or participate in social and professional activities. Previous literature on similar topics, including hip arthroplasty and spinal instrumentation, has also highlighted the psychological burden of security screenings for individuals with metallic implants [3, 5, 6, 9].

The implications of our findings are relevant not only for patients but also for clinicians and policymakers. From a clinical standpoint, it is essential that patients are informed preoperatively about the likelihood of triggering security alarms. Proper counselling can reduce anticipatory anxiety and allow patients to prepare accordingly—for example, by carrying documentation about their implant, arriving earlier at the airport, or requesting alternative screening procedures. Although implant identification cards are unfortunately not universally accepted by airport authorities, they may serve as useful tools for communicating with security staff and facilitating smoother screening experiences [2, 18]. The orthopaedic community should advocate for a global validation of these cards in order to improve patients travel experience. Some patients also reported triggering alarms at other locations including courthouses, federal buildings, stores, amusement parks, and while boarding cruise ships. Patients also reported triggering archway detectors during visits to museums, jails, departments of motor vehicle administration, and entertainment venues [13].

This issue also highlights a gap in airport security protocols. The lack of standardised procedures for dealing with travellers who have medical implants results in wide variability in how these individuals are treated across airports and countries. Some airports may have advanced imaging scanners capable of detecting and differentiating orthopaedic hardware, while others rely solely on metal detection and visual inspections. Some patients have to show their scar to prove they really had a surgery which can be relatively disturbing. The process can be even more bothersome for some genders, certain cultural and mental health conditions due to the potential need for physical contact that may not be deemed appropriate or even possible for certain individuals.

Several limitations should be acknowledged. First, the study relied on self‐reported data, which introduces the possibility of recall bias. However, the nature of the event (triggering a security alarm) is memorable and specific, which likely limits inaccuracies. Second, the study focused on a single type of prosthesis (Stryker® Triathlon), which, although widely used, may not represent all available TKA implants. Variations in implant size, shape, and composition may result in different detection rates. Third, the control group, although matched in terms of demographics, was not randomly selected, which could introduce some selection bias. Finally, the study did not account for airport‐specific differences in security technology or procedures, which may affect generalisability.

Future research could expand upon these findings by examining a broader range of orthopaedic implants, including hip, shoulder, and spinal devices. It would also be valuable to conduct observational studies in airports to assess the real‐time handling of patients with implants and evaluate the effectiveness of identification cards or digital health records in streamlining the screening process. Additionally, exploring the long‐term psychological impact of repeated security screenings on this patient population could inform improvements in both clinical care and airport policy.

## CONCLUSION

This study demonstrates that patients with TKA are significantly more likely to trigger metal detectors during airport security checks compared to individuals without metallic implants. The majority of TKA patients who travelled by air reported repeated alarm activations, and over half found the experience bothersome, highlighting a real impact on quality of life and travel experience.

## AUTHOR CONTRIBUTIONS

Jérôme Murgier formulated the initial project. Etienne Cavaignac developed the theory and undertook the statistical analysis. Régis Pailhé and Jérôme Murgier verified the analytical methods. Jérôme Murgier wrote the paper. All authors discussed the results and contributed to the final manuscript.

## CONFLICT OF INTEREST STATEMENT

Jérôme Murgier is consultant for Arthrex and Microport. Etienne Cavaignac is consultant for Arthrex and Amplitude. Régis Pailhé is consultant for Stryker and Enovis.

## ETHICS STATEMENT

All procedures were performed in accordance with the ethical standards of the institutional and/or national research committee, the 1964 Helsinki Declaration and its later amendments or comparable ethical standards. Informed consent was obtained for each patient in the study. Data collection and analysis were carried out in accordance with the authors' local institutional board. Ethical approval was obtained from the French national ethical committee under the following registration number: n°id‐rcb: 2023‐a00626‐39. Informed consent was obtained from all individual participants included in the study.

## Data Availability

The data that support the findings of this study are available from the corresponding author upon reasonable request.
